# 225. The Immunology and Safety of Maternal RSV Vaccination, Infant Nirsevimab Immunization, or Both Products- Interim Analysis of a Randomized Clinical Trial

**DOI:** 10.1093/ofid/ofaf695.008

**Published:** 2026-01-11

**Authors:** Christina A Rostad, C Mary Healy, Jennifer L Nayak, Lalitha Parameswaran, C Buddy Creech, Judith M Martin, Rebecca C Brady, Catherine Eppes, Kimberly Jones-Beatty, Martina L Badell, Michael Quinn, Mark J Mulligan, Anne-Marie Rick, Katherine Sokolow, Braxton Forde, Vasanthi Avadhanula, Pedro A Piedra, Kalyani Telu, Pratap S Kunwar, Jinjian Mu, Fei Gao, Britta Flach, Marcela Pasetti, Christine M Posavad, Joy Mediema, Cristina Cardemil, James Campbell

**Affiliations:** Emory University School of Medicine and Children's Healthcare of Atlanta, Atlanta, GA; Baylor College of Medicine, Houston, Texas; University of Rochester Medical Center, Rochester, New York; NYU Langone Health, NYU Langone Vaccine Center, New York, New York; Vanderbilt University Medical Center, Nashville, TN; University of Pittsburgh, Pittsburgh, PA; CIncinnati Children's Hospital Medical Center, Cincinnati, Ohio; Baylor College of Medicine, Houston, Texas; University of Maryland, Baltimore, Baltimore, Maryland; Emory University School of Medicine, Atlanta, Georgia; University of Rochester, Rochester, New York; NYU Grossman School of Medicine, New York, New York; University of Pittsburgh, Pittsburgh, PA; VUMC, Franklin, Tennessee; University of Cincinnati, Cincinnati, Ohio; Baylor College of Medicine, Houston, Texas; Baylor College of Medicine, Houston, Texas; EMMES, Chandler, Arizona; The EMMES Company, LLC, GERMANTOWN, Maryland; The Emmes Company, Blacklick, Ohio; Fred Hutchinson Cancer Center, Seattle, Washington; Fred Hutchinson Cancer Research Center, Seattle, Washington; University of Maryland, Baltimore, MD; Fred Hutchinson Cancer Center, Seattle, Washington; FHI 360, Rockville, Maryland; National Institutes of Health, Rockville, Maryland

## Abstract

**Background:**

RSV is the leading cause of lower respiratory tract infections (LRTIs) in infants. Although both maternal RSVpreF vaccination and infant nirsevimab immunization have been approved for the prevention of RSV LRTIs, the two products have not been evaluated in a single study, nor has their sequential administration been studied systematically.

**Figure:**
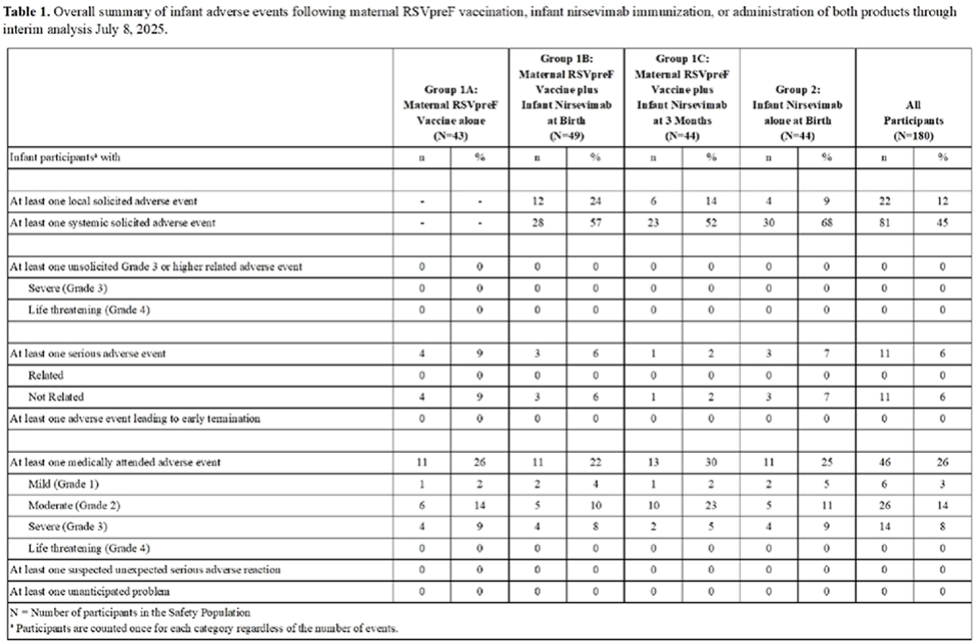

**Figure:**
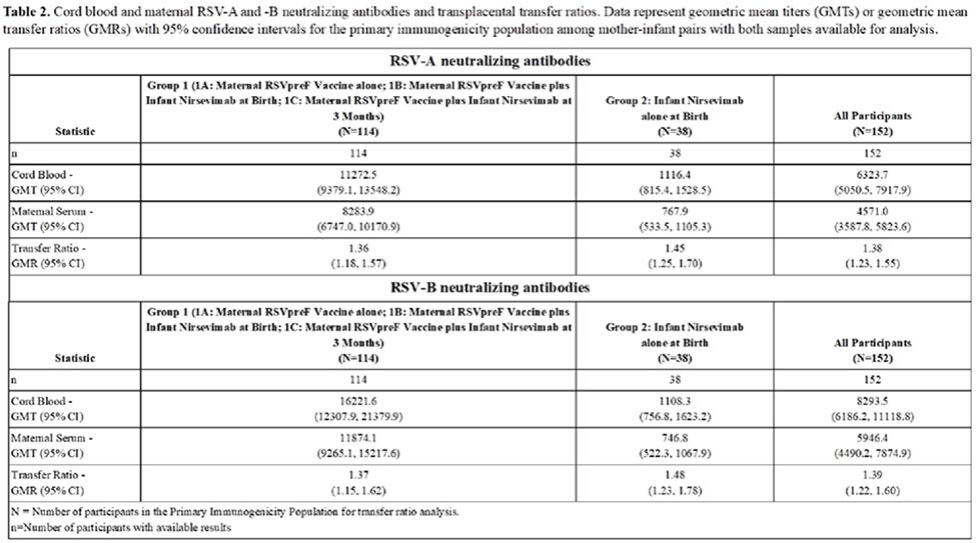

**Methods:**

We performed a prospective, randomized, open-label, Phase 4 study at 8 US sites of mother-infant pairs randomized 1:1:1:1 during pregnancy into four groups: Group 1A: maternal RSVpreF vaccine alone; Group 1B: maternal RSVpreF vaccine/infant nirsevimab at birth; Group 1C: maternal RSVpreF vaccine/infant nirsevimab at 3 months; or Group 2: infant nirsevimab alone at birth. We are following the mother-infant pairs for 12 months to ascertain safety, infant tolerability, and the magnitude and durability of RSV-A and -B neutralizing antibodies (nAbs). These results represent the interim analysis of participants from September 19, 2024, to July 8, 2025, which included up to 4-month infant follow-up.

**Figure 1.**
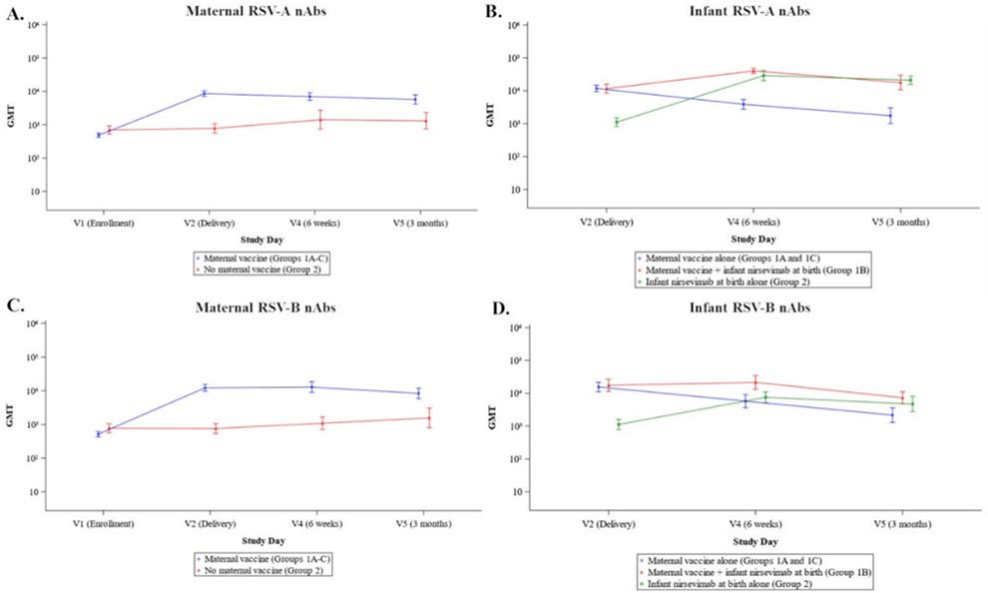
RSV-A and -B neutralizing antibodies in mothers and infants following maternal RSVpreF vaccination, infant nirsevimab administration at birth, or administration of both products. Data represents geometric mean titers (GMTs) with 95% confidence intervals by study group and time point for the primary immunogenicity population.

**Results:**

181 mother-infant pairs were enrolled. Both products alone and in combination were safe and no related SAEs were observed in mothers or infants (Table 1). RSV-preF vaccination boosted maternal RSV-A nAb titers 17.35-fold at the time of delivery, and titers were durable through 3 months post-delivery (Figure 1A). The geometric mean transfer ratio (GMR) of RSV-A nAbs was >1.3 and similar across groups (Table 2). For infants of mothers who received RSVpreF vaccine alone (Groups 1A and 1C at the time of interim analysis), RSV-A nAbs peaked at delivery and gradually declined over 3 months (Figure 1B). Administration of nirsevimab to infants at birth increased RSV-A nAbs 3.53-fold in those whose mothers had received RSVpreF vaccine (Group 1B) and 25.12-fold in those who had not (Group 2) after 6 week follow-up. Infants who received nirsevimab at birth (Groups 1B and 2) had similar peak nAb titers regardless of maternal vaccination status (Figure 1B). Similar trends were observed for RSV-B nAbs (Figure 1C and D).

**Conclusion:**

Maternal RSVpreF vaccine and infant nirsevimab administration, either alone or in combination, were safe and elicited high RSV nAb titers in mothers and infants through interim follow-up.

**Disclosures:**

All Authors: No reported disclosures

